# Multimodal Rehabilitation with Focal Vibration After Botulinum Toxin Injection in Ambulatory Children with Cerebral Palsy: A Proof-of-Concept Pilot Study

**DOI:** 10.3390/toxins18080327

**Published:** 2026-07-28

**Authors:** Alessandro Picelli, Rita Di Censo, Ilaria Di Maria, Antonella Vangelista, Maria Vittoria Benetti, Nicola Smania, Valentina Varalta, Mirko Filippetti

**Affiliations:** 1 Department of Neurosciences, Biomedicine and Movement Sciences, University of Verona, 37134 Verona, Italy; ilaria.dimaria@univr.it (I.D.M.); mariavittoria.benetti@gmail.com (M.V.B.); nicola.smania@univr.it (N.S.); valentina.varalta@univr.it (V.V.); mirko.filippetti@univr.it (M.F.); 2 Department of Neurosciences, University Hospital of Verona, 37126 Verona, Italy; rita.dicenso@aovr.veneto.it; 3 Canadian Advances in Neuro-Orthopedics for Spasticity Consortium (CANOSC), Kingston, ON K7K 1Z6, Canada; 4 Poliambulanza Foundation, 25124 Brescia, Italy; antonella.vangelista@poliambulanza.it

**Keywords:** botulinum toxins, muscle spasticity, rehabilitation

## Abstract

Cerebral palsy is a leading cause of childhood motor disability, and ambulatory children with spastic hemiplegia commonly present with dynamic equinus, calf muscle overactivity, and gait impairment. Evidence on focal vibration after botulinum toxin type A injection remains limited. This exploratory proof-of-concept pilot study assessed short-term changes following a four-week multimodal rehabilitation program incorporating focal vibration. In this uncontrolled single-group pre–post study, nine ambulatory children (mean age, 12.1 years), all classified as level II on the Gross Motor Function Classification System, received onabotulinumtoxinA injections into the affected gastrocnemius muscles, followed by twelve outpatient sessions combining focal vibration, conventional physiotherapy, and robotic gait training. Passive ankle dorsiflexion increased, calf muscle spasticity decreased, and selected gait parameters improved. Walking speed increased from 0.79 to 0.90 m per second, an absolute change of 0.11 m per second, comparable in magnitude to published estimates of clinically important change in ambulatory children with cerebral palsy. After Bonferroni correction, only walking speed and affected-limb stride length remained significant. No adverse events occurred. These findings describe outcomes associated with the combined program but do not establish the efficacy or independent contribution of focal vibration.

## 1. Introduction

Cerebral palsy (CP) is a leading cause of motor disability in childhood, with a global prevalence of 1.6–3.0 cases per 1000 live births [1,2]. Spastic hemiplegic CP is characterized by predominantly unilateral motor impairment. Foot and ankle deformities are common in ambulatory children with this condition and may adversely affect gait efficiency, orthotic management, and overall mobility [1]. Equinus is among the most frequent deformity patterns [2]. The associated gait dysfunction reflects not only plantar-flexor overactivity but also impaired selective motor control, muscle weakness, altered biomechanics, and reduced balance and walking efficiency [1,2].

Botulinum toxin type A (BoNT-A) is a cornerstone of focal spasticity management in children with CP and is widely used when lower-limb muscle overactivity, particularly dynamic equinus, interferes with gait and function [3,4,5,6]. By producing a temporary, targeted reduction in muscle overactivity, BoNT-A may decrease spasticity and improve ankle passive range of motion (PROM) [6,7]. However, reducing muscle overactivity alone does not necessarily produce proportional gains in voluntary motor control, gait, or functional performance. The response to BoNT-A depends on several factors, including patient selection, treatment goals, injection technique, muscle targeting, and post-injection rehabilitation [3,4,5,6,7,8]. Adjunctive interventions aimed at improving muscle extensibility, selective motor activation, motor relearning, and task-specific performance may therefore help optimize the post-injection therapeutic window [7,8].

Post-injection management in children with CP has consequently been investigated within a multimodal rehabilitation framework. Depending on the clinical presentation and therapeutic goals, BoNT-A has been combined with conventional physiotherapy, stretching, orthotic management, serial casting, electrical or neuromuscular stimulation, and intensive task-specific training [7,8,9]. In upper-limb rehabilitation, it has also been combined with occupational therapy, constraint-induced movement therapy, and bimanual training to promote active use of the affected limb [10]. Although these upper-limb approaches are not directly comparable with lower-limb gait rehabilitation, they illustrate the broader principle that temporary reductions in muscle overactivity should be paired with interventions intended to translate changes in tone into motor learning and functional gains. The comparative effectiveness of these adjunctive strategies remains uncertain; however, because treatment protocols, study populations, and outcome measures vary substantially.

Focal muscle vibration has emerged as a potential neuromodulatory adjunct within this framework [9]. High-frequency mechanical vibration applied to a selected muscle or tendon provides intensive proprioceptive stimulation, particularly through activation of muscle-spindle afferents, and may modulate spinal and supraspinal sensorimotor circuits [11,12]. This afferent input may facilitate activation of the stimulated muscle while influencing antagonist activity through reciprocal inhibitory pathways mediated by Ia inhibitory interneurons [11,12]. Thus, vibration of the tibialis anterior, an antagonist of the ankle plantar flexors, may facilitate dorsiflexor recruitment and promote reciprocal inhibition of the gastrocnemius–soleus complex. The tibialis anterior and peroneal muscles were selected as targets on the basis of the pathophysiology of equinus and equinovarus gait, in which excessive plantar-flexor and invertor activity may coexist with insufficient recruitment of the dorsiflexors and evertors. This agonist–antagonist imbalance can compromise foot clearance and dynamic ankle stability. Accordingly, BoNT-A may reduce excessive plantar-flexor activity at the neuromuscular junction, whereas focal vibration may enhance proprioceptive input, selective activation of muscles involved in ankle control, and agonist–antagonist coordination [9]. These mechanisms nevertheless remain hypothetical in children with CP and require direct neurophysiological confirmation.

Clinical evidence on focal vibration remains preliminary but is not limited to its combined use with BoNT-A in pediatric CP. In adults with stroke, focal muscle vibration and localized vibrotactile stimulation have shown potential effects on spasticity, voluntary motor performance, and sensorimotor function [13]. These findings support the feasibility and neurophysiological plausibility of localized vibration-based interventions, although differences in stimulation parameters, target muscles, treatment dose, and outcome measures limit comparisons across studies. Vibrotactile stimulation has also been investigated as a means of enhancing sensory input and use of the affected limb during stroke rehabilitation. Pediatric evidence is more limited. Preliminary studies have examined focal muscle vibration in small samples of children with CP, and localized vibrotactile devices have been explored to increase awareness and use of the affected upper limb in unilateral CP [14]. Evidence from other pediatric neurological populations is particularly sparse and heterogeneous and often concerns sensory or vibrotactile stimulation rather than standardized focal muscle vibration protocols. Findings from adult stroke and related pediatric neurorehabilitation applications therefore provide useful mechanistic and clinical context but cannot be directly extrapolated to ambulatory children with spastic hemiplegic CP.

Most studies of vibration-based interventions in children with CP have focused on whole-body vibration, with potential benefits reported for spasticity, muscle performance, balance, and selected gait outcomes; however, protocols are heterogeneous and findings remain inconclusive [15,16,17,18,19,20]. Unlike whole-body vibration, focal vibration can be directed at muscles selected according to the individual motor impairment, thereby limiting nonspecific whole-body mechanical exposure. It can also be delivered concurrently with passive mobilization or active-assisted exercise, facilitating integration into a multimodal rehabilitation session. These features may make focal vibration particularly suitable for targeting ankle muscle activation and agonist–antagonist coordination in children with dynamic equinus. Nevertheless, evidence on focal vibration after BoNT-A injection remains scarce. A small preliminary pediatric study reported possible reductions in spasticity following focal muscle vibration [14], while a recent case report involving a child with unilateral spastic CP described improvements in walking speed, plantar-flexor overactivity, passive muscle extensibility, and pain after combined focal vibration and BoNT-A treatment [21]. Although these observations are insufficient to establish efficacy or determine the independent contribution of focal vibration, they support further investigation within structured multimodal rehabilitation programs.

Against this background, the present study examined whether a four-week multimodal rehabilitation program incorporating focal vibration after BoNT-A injection was associated with short-term changes in ankle dorsiflexion, calf muscle spasticity, and walking performance in ambulatory children with spastic hemiplegic CP. We hypothesized that the program would increase passive ankle dorsiflexion, shift active ankle dorsiflexion toward neutral, reduce calf muscle AS scores, increase walking speed, affected-limb stride length, and affected-limb single-support time, and decrease double-support time. Given the small sample, single-group pre–post design, and combined intervention, the study was conceived as an exploratory, hypothesis-generating proof-of-concept investigation rather than a confirmatory trial. It was not designed to establish causality or isolate the contribution of focal vibration from those of BoNT-A, conventional physiotherapy, and robotic gait training.

## 2. Results

Nine participants were included in the analysis. Their mean age was 12.1 years (SD 3.1); seven were female, and eight had right-sided hemiplegia. All participants were classified as level II on both the Gross Motor Function Classification System (GMFCS) and the Manual Ability Classification System (MACS) [22]. At baseline, six participants had a calf muscle Ashworth Scale (AS) score of 2, and three had a score of 3 [23]. None had severe cognitive impairment or unstable behavioral factors, and none were receiving systemic muscle relaxants or intrathecal baclofen therapy at enrollment. No adverse events or other treatment-related complications were observed or reported during the intervention, including pain or skin irritation related to focal vibration or falls during robotic gait training.

Shapiro–Wilk tests of the within-participant change scores indicated departures from normality for affected-limb single-support duration, double-support time expressed both as a percentage of the gait cycle and in seconds, and affected-limb stance-phase duration (all *p* < 0.05). No statistically significant departures from normality were identified for the remaining continuous outcomes. Given the small sample and the non-normal distribution of several change scores, nonparametric methods were applied consistently across outcomes.

Changes in clinical and gait outcomes from baseline (T0) to the post-treatment assessment (T1) are presented in Table 1, together with absolute and percentage changes, 95% confidence intervals, matched-pairs rank-biserial correlation effect sizes, unadjusted *p*-values, and false discovery rate-adjusted q-values. At the nominal significance level, ankle dorsiflexion PROM increased, while calf muscle spasticity, assessed using the AS, decreased from T0 to T1. Ankle dorsiflexion active range of motion (AROM) changed from −17.4° at T0 to −12.6° at T1, but the difference was not statistically significant. Negative AROM values indicate plantarflexion relative to the neutral position, defined as 0°; thus, the observed change represents movement toward neutral and a reduction in the active plantarflexion deficit rather than “negative dorsiflexion.”

Among the gait outcomes, walking speed and affected-limb stride length increased significantly. Affected-limb single-support time also increased, both as a percentage of the gait cycle and in seconds, whereas double-support time decreased in both formats. No significant changes were observed in cadence or affected-limb stance-phase duration. After Benjamini–Hochberg correction across the 11 outcomes, PROM, AS score, walking speed, affected-limb stride length, both measures of affected-limb single-support time, and both measures of double-support time remained statistically significant. Under the more conservative Bonferroni correction, only walking speed and affected-limb stride length remained significant. The other findings should therefore be interpreted as exploratory.

Leave-one-out sensitivity analyses generally confirmed the direction of the principal findings. The affected-limb single-support duration result was sensitive to the exclusion of individual participants: it became nonsignificant in six of the nine iterations (*p* = 0.0625) and remained significant in three (*p* = 0.0313). This finding should therefore be considered statistically fragile.

**Figure 1:**
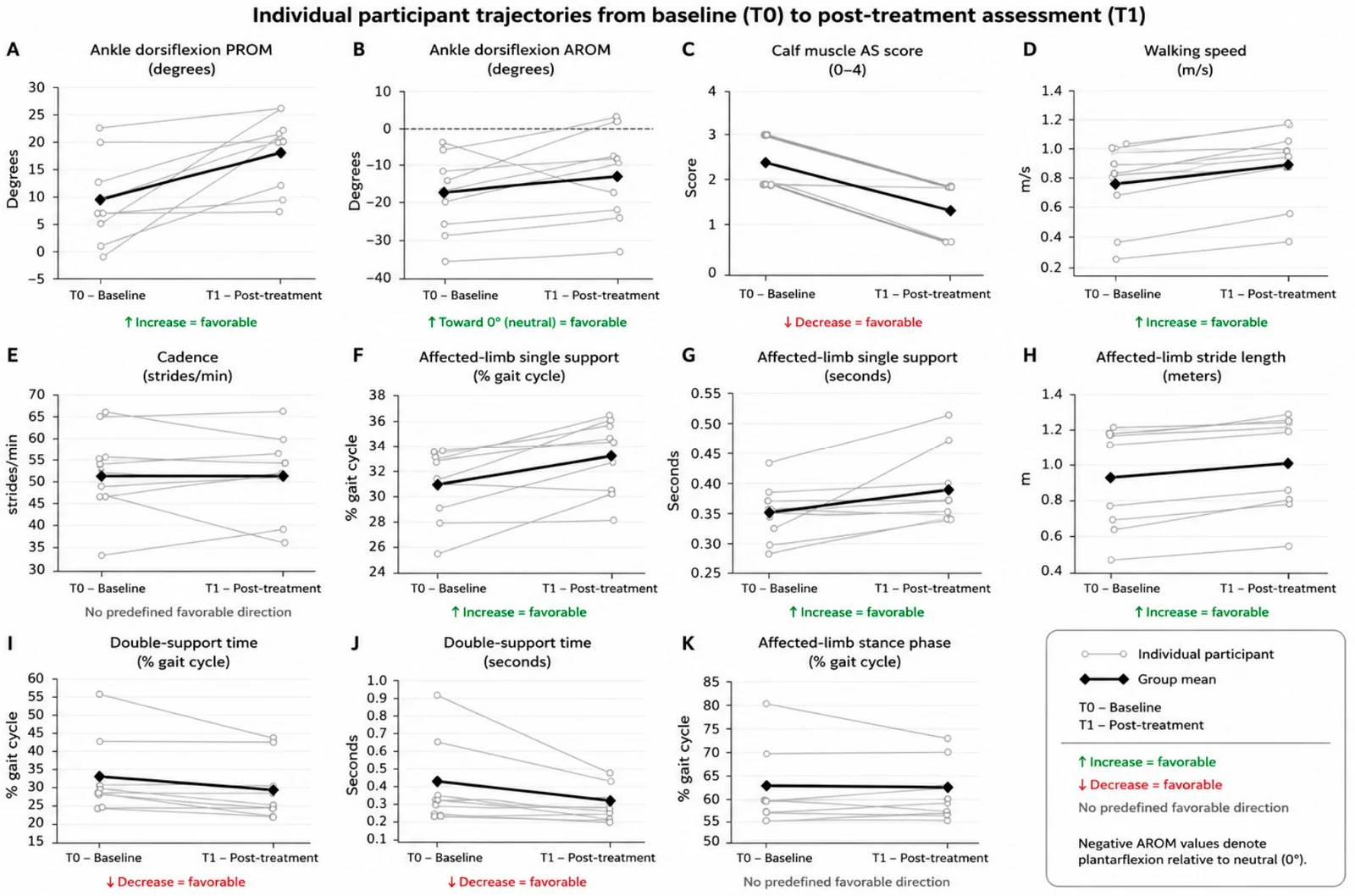
Individual participant trajectories from baseline to the post-treatment assessment. Each panel shows individual values at baseline (T0) and after treatment (T1), together with the group mean, for: passive ankle dorsiflexion range of motion (**A**); active ankle dorsiflexion range of motion (**B**); calf muscle Ashworth Scale score (**C**); walking speed (**D**); cadence (**E**); affected-limb single-support time expressed as a percentage of the gait cycle (**F**); affected-limb single-support time expressed in seconds (**G**); affected-limb stride length (**H**); double-support time expressed as a percentage of the gait cycle (**I**); double-support time expressed in seconds (**J**); and affected-limb stance-phase duration expressed as a percentage of the gait cycle (**K**). Favorable change corresponds to increased passive ankle dorsiflexion, active ankle dorsiflexion toward the neutral position, walking speed, affected-limb stride length, and affected-limb single-support time, and to decreased Ashworth Scale score and double-support time. No prespecified favorable direction was assigned to cadence or affected-limb stance-phase duration.

Exploratory Spearman analyses identified no statistically significant associations between age and changes in the clinical or gait outcomes. The correlation coefficients were as follows: PROM, ρ = −0.31 (*p* = 0.418); AROM, ρ = 0.28 (*p* = 0.460); AS score, ρ = 0.14 (*p* = 0.718); walking speed, ρ = −0.15 (*p* = 0.693); cadence, ρ = 0.61 (*p* = 0.084); affected-limb single-support percentage, ρ = −0.24 (*p* = 0.535); affected-limb single-support duration, ρ = −0.64 (*p* = 0.065); affected-limb stride length, ρ = −0.55 (*p* = 0.127); double-support percentage, ρ = 0.15 (*p* = 0.700); double-support duration, ρ = 0.40 (*p* = 0.284); and affected-limb stance-phase duration, ρ = −0.10 (*p* = 0.793). The largest associations were observed for affected-limb single-support duration, cadence, and stride length. Given the small sample and broad age range, these analyses were underpowered and should be regarded as descriptive. Individual participant trajectories are shown in Figure 1.

## 3. Discussion

At the nominal significance level, this proof-of-concept pilot study found that a multimodal post-injection rehabilitation program incorporating focal vibration was followed by increased ankle dorsiflexion PROM, reduced calf muscle spasticity, and improvements in selected spatiotemporal gait parameters. AROM improved numerically but not significantly. The robustness of the findings varied according to the method used to account for multiple comparisons. PROM, AS score, walking speed, affected-limb stride length, single-support time, and double-support time remained significant after false discovery rate correction, whereas only walking speed and affected-limb stride length remained significant after the more conservative Bonferroni correction. The findings for PROM, muscle tone, and support-time variables should therefore be considered exploratory and hypothesis-generating rather than confirmatory.

The timing and direction of the changes in PROM and muscle tone are compatible with the expected therapeutic effects of BoNT-A. Reductions in plantar-flexor overactivity and improvements in passive ankle mobility are commonly observed during the first weeks after injection [3,4,5,6,7,8], and the post-treatment assessment occurred within this therapeutic window. In the absence of a BoNT-A-only control group, the improvements in PROM and AS score could therefore be explained partly or entirely by BoNT-A itself. The present design does not demonstrate that focal vibration, conventional physiotherapy, or robotic gait training provided an incremental benefit beyond the injection. Rather, the findings describe the response to the combined post-injection program as a whole, and any proposed additive or synergistic effect of focal vibration remains hypothetical.

A plausible neurophysiological rationale nevertheless supports the inclusion of focal vibration in multimodal rehabilitation. High-frequency vibration applied to a muscle or tendon activates muscle-spindle afferents, particularly group Ia fibers, and may modulate spinal and supraspinal sensorimotor circuits [11,12]. Sustained afferent input may facilitate activation of the vibrated muscle through mechanisms related to the tonic vibration reflex and may influence antagonist activity through reciprocal inhibitory pathways mediated by Ia inhibitory interneurons [11,12]. The tibialis anterior was selected because it is the principal ankle dorsiflexor and functionally opposes the overactive plantar flexors involved in equinus gait. The peroneal muscles were targeted because they contribute to eversion and mediolateral ankle control and may help counter the plantarflexion and inversion tendencies associated with equinus or equinovarus patterns.

From this perspective, BoNT-A and focal vibration could act on complementary components of ankle motor control. BoNT-A reduces excessive plantar-flexor activity at the neuromuscular junction, potentially decreasing resistance to dorsiflexion, whereas vibration of the tibialis anterior and peroneal muscles may increase proprioceptive input and facilitate recruitment of muscles involved in dorsiflexion, eversion, and dynamic ankle stability. Passive mobilization and active-assisted exercise during vibration may further exploit the temporary reduction in plantar-flexor resistance. One may speculate that these combined effects create more favorable biomechanical and sensorimotor conditions for ankle movement and subsequent gait practice; however, this possibility was not directly tested. No electromyographic, reflex, or other neurophysiological measures were collected; consequently, activation of the tonic vibration reflex, reciprocal inhibition, enhanced agonist drive, or an interaction between BoNT-A and focal vibration cannot be inferred from the clinical outcomes.

Robotic gait training may also have contributed by providing intensive, repetitive, task-specific practice of the gait cycle [24]. Repeated stepping may promote more consistent temporal organization of stance and swing, facilitate loading of the affected limb, and reinforce locomotor patterns under controlled conditions. Delivering robotic gait training immediately after vibration and physiotherapy may have enabled participants to practice walking under the conditions created by the preceding treatment phase, although this interpretation is speculative. Because all participants received focal vibration with physiotherapy followed by robotic gait training, the effects of the individual components, their interactions, and any order effects cannot be distinguished. The fixed sequence also precludes conclusions about whether robotic training after vibration is preferable to another treatment order.

The absence of a statistically significant improvement in AROM warrants specific consideration. Negative AROM values indicate that participants remained in plantarflexion relative to the neutral ankle position, defined as 0°. Thus, the change from −17.4° at baseline to −12.6° after treatment represents movement toward neutral and a reduction in the active plantarflexion deficit rather than “negative dorsiflexion.” Active dorsiflexion, however, depends not only on reduced plantar-flexor overactivity but also on dorsiflexor strength, recruitment timing, and selective motor control. Selective motor control is the ability to isolate voluntary movement at an individual joint without obligatory mass flexor or extensor patterns and is a major determinant of gait performance in children with CP [2]. It reflects impaired central motor organization and is not necessarily restored by reducing peripheral muscle overactivity with BoNT-A. Similarly, although focal vibration may transiently modulate sensory input and motor excitability, it cannot be assumed to normalize selective motor control. The absence of a direct measure of selective motor control limits interpretation of the AROM findings and should be addressed in future studies.

The gait findings also warrant cautious interpretation. Walking speed and affected-limb stride length were the only outcomes that remained statistically significant after Bonferroni correction and thus represent the most robust findings. Mean walking speed increased from 0.79 to 0.90 m/s, corresponding to an absolute change of 0.11 m/s and a relative increase of 14.0%. Oeffinger et al. reported GMCS-specific estimates of clinically important change in walking velocity, expressed relative to age-matched normative values, including an estimate of approximately 10.9% for children classified as level II [25]. The relative increase in the present cohort was therefore similar in magnitude. This comparison should nonetheless be interpreted cautiously because the published estimate was not a universal absolute threshold of 0.10 m/s and was derived using a different method of expressing walking velocity. Its applicability may also vary with age, walking test, measurement context, and other participant characteristics. Moreover, the uncontrolled design prevents the observed change from being interpreted as evidence of a clinically important treatment effect. Individual trajectories should also be considered (Figure 1), because a potentially meaningful group-level change does not imply a comparable response in every participant.

Affected-limb single-support time increased and double-support time decreased after false discovery rate correction, but these findings did not remain significant after Bonferroni correction. The changes may be consistent with greater loading of the affected limb and less reliance on bilateral support, potentially reflecting a more stable and efficient walking pattern. Gait in children with CP is multifactorial, however, and is influenced by selective motor control, proximal muscle function, balance, compensatory strategies, lever-arm dysfunction, and established locomotor patterns [2]. The BoNT-A-mediated reduction in plantar-flexor overactivity could itself facilitate foot progression and loading during gait [4]. The present findings therefore cannot establish whether changes in support times resulted from focal vibration, robotic task practice, BoNT-A, or their combined effects. The absence of significant changes in cadence and affected-limb stance-phase duration further suggests that the response was selective rather than uniform across gait domains.

Effect sizes, confidence intervals, and percentage changes from baseline were reported to characterize the magnitude and direction of the observed changes, but these measures do not directly establish clinical importance. Minimal clinically important differences were not prespecified and have not been consistently established for all outcomes in this population. Participant-level data also showed variability in the magnitude and, for some outcomes, the direction of change. Group-level estimates should therefore be interpreted cautiously in this small, heterogeneous sample.

The findings should be considered in the context of the limited literature on focal vibration in pediatric CP. Most vibration studies in this population have examined whole-body vibration, using heterogeneous protocols and yielding inconclusive results for spasticity, muscle performance, balance, and gait [15,16,17,18,19,20]. Evidence on focal muscle vibration is more limited. A recent case report of focal vibration therapy combined with BoNT-A injection in a child with unilateral spastic CP described improvements in walking speed, plantar-flexor overactivity, passive muscle extensibility, and pain [21]. Although direct comparison is limited by the single-case design and differences in treatment protocols, that report and the present exploratory observations support further investigation of focal vibration within structured post-BoNT-A rehabilitation. Controlled designs are required to distinguish the effects of BoNT-A from those of focal vibration, physiotherapy, and robotic gait training.

### Study Limitations

This proof-of-concept pilot study has several important limitations. First, the small sample, single-center setting, and uncontrolled single-group pre–post design limit the precision and generalizability of the estimates and preclude causal inference. Without BoNT-A-only, usual-care, sham-vibration, or no-treatment control groups, the observed changes cannot be distinguished from the expected effects of BoNT-A, placebo or expectation effects, regression to the mean, normal within-participant variability, or other time-related changes. Second, the clinicians who performed the T0 and T1 assessments were not blinded to treatment exposure. Observer and expectation bias therefore cannot be excluded, particularly for clinician-administered outcomes such as goniometric ankle measurements and the AS. Although standardized assessment procedures were used and the AS has established reliability in children with CP [23], study-specific intra-rater and inter-rater reliability were not evaluated. Reliability of the goniometric measurements was also not assessed in this sample. Measurement error may therefore have contributed to the observed changes, particularly because measurements were recorded in 5° increments. Third, all participants received the same fixed sequence: focal vibration combined with physiotherapy, followed by robotic gait training. The contributions of vibration, concurrent physiotherapy, robotic practice, and treatment sequencing therefore cannot be separated. Carryover and order effects are also possible because robotic gait training was always delivered immediately after vibration rather than in a randomized or counterbalanced sequence. Fourth, the broad age range may have introduced developmental heterogeneity in baseline gait characteristics and treatment response. Although exploratory analyses found no statistically significant associations between age and outcome changes, they were markedly underpowered. The laterality distribution was also imbalanced: eight participants had right-sided hemiplegia and one had left-sided hemiplegia. This imbalance precluded meaningful side-stratified analyses and may have introduced additional unmeasured heterogeneity. Fifth, selection bias is possible. Children and families who were willing and able to attend 12 treatment sessions over four weeks may have been more motivated, had greater family support, or faced fewer logistical barriers than the broader population of ambulatory children with spastic hemiplegic CP. The findings may therefore not generalize to children with different functional levels, comorbidities, adherence capacity, or access to intensive rehabilitation. Sixth, the number of outcomes increased the risk of type I error. Although false discovery rate and Bonferroni sensitivity analyses were performed, several findings that were significant at the nominal level did not remain significant under the more conservative Bonferroni procedure and should therefore be regarded as exploratory. Moreover, because the study was not prospectively registered and no publicly accessible protocol or statistical analysis plan was available, it is not possible to verify independently that all 11 outcomes and corresponding analyses were prespecified before data analysis. This limitation is particularly relevant to multiple testing and the potential for selective outcome reporting. Future confirmatory trials should be prospectively registered and should prespecify a primary outcome, secondary outcomes, and the statistical analysis plan. Minimal clinically important differences were also not prespecified and are not established for all outcomes; statistical effect magnitude should therefore not be equated directly with clinical relevance. Finally, participants were assessed only at baseline and immediately after the four-week intervention. No intermediate or longer-term follow-up was performed, and the durability of the observed changes is unknown. These findings should therefore be considered hypothesis-generating and require confirmation in larger, adequately powered, prospectively registered, assessor-blinded controlled trials with prespecified primary outcomes, appropriate comparator groups, randomized or factorial intervention components, study-specific reliability procedures, and longer follow-up.

## 4. Conclusions

This proof-of-concept pilot study documents short-term changes in ankle dorsiflexion PROM, calf muscle spasticity, and selected spatiotemporal gait parameters following a multimodal post-BoNT-A rehabilitation program incorporating focal vibration in ambulatory children with spastic hemiplegic CP. Because the intervention also included conventional physiotherapy and robotic gait training and was evaluated using an uncontrolled single-group pre–post design, the study does not establish the efficacy or independent contribution of focal vibration. The findings should therefore be regarded as exploratory and hypothesis-generating. A larger randomized controlled trial comparing the multimodal program with a BoNT-A-only control condition, with blinded outcome assessment and longer-term follow-up, is needed to determine whether focal vibration provides additional and sustained clinical benefit. If efficacy is confirmed, subsequent studies should investigate optimal vibration frequency, amplitude, dose, target muscles, and treatment sequence, as well as the durability of effects beyond the immediate post-treatment period.

## 5. Materials and Methods

### 5.1. Study Design

This single-center, exploratory proof-of-concept pilot study used an uncontrolled single-group pre–post design and was conducted in an outpatient setting between January and October 2019. The study was not prospectively registered in a public clinical trial registry because it was conceived as a small, feasibility-oriented investigation intended to generate preliminary estimates rather than confirmatory evidence of efficacy. The absence of prospective registration is acknowledged as a methodological limitation. No formal sample-size calculation was performed. The target sample of nine participants was determined pragmatically on the basis of feasibility, the expected availability of eligible patients during the recruitment period, and the clinical and organizational resources required to complete the intervention and assessments. The study was therefore not powered to establish treatment efficacy.

The inclusion criteria were age 7–16 years; a diagnosis of spastic hemiplegic CP; classification as level II on the GMFCS and MACS [22]; an AS score ≥ 2 in the affected calf muscles [23]; no focal treatment for spasticity with BoNT-A during the 5 months before enrollment; and the ability to walk barefoot independently for at least 10 m without an assistive device. The AS threshold was selected to identify clinically relevant calf muscle spasticity and reflected the routine criteria used at our center when considering BoNT-A treatment. The 5-month interval since the previous BoNT-A treatment was chosen to minimize carryover from earlier injections and to ensure that baseline assessment occurred after the expected clinical effects of the preceding treatment had substantially diminished.

The exclusion criteria were participation in another clinical trial; severe cognitive impairment; unstable behavioral factors that could interfere with assessment or treatment; fixed contractures or bony deformities of the lower limbs; previous neurolytic treatment for calf muscle spasticity; previous orthopedic ankle surgery, including tendon lengthening or transfer, tenotomy, muscle release, or arthrodesis; previous neurosurgical procedures, including lower-limb selective neurectomy or selective dorsal rhizotomy; concomitant neurological or orthopedic conditions affecting the lower limbs; contraindications to BoNT-A administration; ongoing treatment with systemic muscle relaxants or an intrathecal baclofen pump; active infection; and documented or suspected fractures. All participants and/or their legal guardians provided written informed consent, as appropriate. The study was approved by the local Institutional Review Board and conducted in accordance with the Declaration of Helsinki.

All participants received onabotulinumtoxinA injections (AbbVie, Irvine, CA, USA) into the spastic muscles of the affected lower limb. The medial and lateral heads of the gastrocnemius on the affected side were targeted. In accordance with the Italian prescribing information, the dose was 4 U/kg, up to a maximum total dose of 200 U [26]. Injection sites were identified using anatomical landmarks and ultrasound guidance [27,28,29]. After application of a topical local anesthetic cream (EMLA 5%; AstraZeneca, London, UK), onabotulinumtoxinA was administered at a concentration of 100 U/mL after dilution with 0.9% saline.

### 5.2. Clinical and Walking Assessment

Participants were assessed at baseline, before treatment, and after completion of the intervention. Clinical outcomes included ankle dorsiflexion PROM and AROM of the affected limb and calf muscle spasticity assessed using the AS [23]. Maximum ankle dorsiflexion was measured with the knee extended using a handheld goniometer and recorded to the nearest 5°. Dorsiflexion angles were assigned positive values and plantarflexion angles negative values, with 0° representing the neutral joint position.

Only the AS was used to assess calf muscle tone. No additional measure was administered because the feasibility-oriented proof-of-concept design required a limited clinical assessment battery. The AS is a 5-point ordinal scale grading resistance of a relaxed limb to rapid passive stretch: 0 = no increase in muscle tone; 1 = slight increase in tone at the end of the range of motion; 2 = more marked increase in tone through most of the range; 3 = considerable increase in tone; and 4 = the affected part is rigid in flexion or extension.

Walking performance was assessed using the GAITRite system (GAITRite Gold, version 3.2b; CIR Systems, Sparta, NJ, USA), a pressure-sensitive electronic walkway that provides objective spatiotemporal gait parameters [30]. To minimize the effects of acceleration and deceleration, participants walked along the 7.66 m walkway within a 12 m course. Three trials were recorded, and their mean was used for analysis. Walking speed was reported in raw meters per second and was not normalized to leg length. Participants were tested under their customary walking conditions, including footwear, orthoses, or assistive devices when routinely required. The same conditions were maintained at both assessments to ensure within-participant consistency and safety. Outcomes of interest were walking speed, cadence, stride length, single-support time, double-support time, and affected-limb stance-phase duration.

### 5.3. Intervention

The rehabilitation program began 7 days after BoNT-A injection. All participants completed twelve individual 40 min sessions, delivered three times per week for four consecutive weeks. Each session comprised two consecutive phases.

During the first phase, focal vibration was delivered using the Vibra 3.0 device (AD Swiss MedTech SA, Gravesano, Switzerland). The applicator was positioned over the myotendinous regions of the tibialis anterior and peroneal muscles of the affected limb while the participant lay supine. Vibration was applied for 20 min at a frequency of 150 Hz and an amplitude of 1 mm.

Concurrent physiotherapy was delivered on a treatment table in the rehabilitation gym, with each participant supine and treated individually at a 1:1 therapist-to-patient ratio. The standardized content comprised passive ankle dorsiflexion mobilization and active-assisted ankle dorsiflexion and eversion exercises performed within the available pain-free range. Participants completed as many repetitions as tolerated during the 20 min vibration phase; the number of repetitions was neither predefined nor standardized. During the second phase, participants underwent 20 min of robotic gait training [24].

### 5.4. Statistical Analysis

Statistical analyses followed a single-group pre–post design. For each continuous outcome, normality of the within-participant change scores was assessed separately using the Shapiro–Wilk test and visual inspection of the distribution and quantile–quantile plots. Normality was therefore evaluated on an outcome-specific basis rather than through a single global assessment.

Although the Shapiro–Wilk test did not reject normality for every continuous variable, the sample of nine participants limited its ability to establish distributional adequacy. Several change-score distributions departed significantly from normality, and some variables contained discrete values, ties, or limited variation. Two-sided Wilcoxon signed-rank tests were therefore used for all paired comparisons, including continuous gait outcomes, to avoid reliance on unstable parametric assumptions and to maintain a consistent analytical framework. The AS was analyzed nonparametrically because it is ordinal. Continuous variables were summarized as mean and standard deviation, whereas AS scores were reported as median and interquartile range.

For each comparison, matched-pairs rank-biserial correlation (*r*rb) was calculated as the effect-size measure. Mean paired changes were also reported with bias-corrected and accelerated bootstrap 95% confidence intervals based on 100,000 resamples. Group-level relative change from baseline was calculated as 100 × (T1 mean − T0 mean)/|T0 mean|. Because the AS is ordinal, its percentage change was reported for descriptive purposes only and should be interpreted cautiously.

Given the exploratory design, unadjusted *p*-values were retained as the primary analyses. Robustness to multiple testing was examined using both the Bonferroni correction and the Benjamini–Hochberg false discovery rate procedure across all 11 outcomes. The influence of individual participants was assessed through leave-one-out analyses, in which the Wilcoxon signed-rank tests were repeated after sequentially excluding each participant. A per-protocol analysis included participants who completed the intervention and both T0 and T1 assessments. Because all nine participants completed the treatment program and had complete outcome data, the per-protocol and complete-case populations were identical, and no imputation was required. No observation was excluded solely because of statistical magnitude in the absence of evidence of measurement or data-entry error. Exploratory Spearman rank correlations assessed associations between age and within-participant changes in each clinical and gait outcome. All tests were two-sided, with statistical significance set at *p* < 0.05.

## Figures and Tables

**Table 1:** Changes in clinical and gait outcomes from before (T0) to after treatment (T1).

Outcome	T0	T1	95% CI	Change	*r*rb	*p*	FDR *q*
Ankle dorsiflexion PROM (deg)	10.0 (7.5)	18.2 (6.3)	8.2 (3.9 to 13.9)	+82.2%	1.00	0.0156	0.0286
Ankle dorsiflexion AROM (deg)	−17.4 (10.7)	−12.6 (11.5)	4.9 (−2.4 to 8.6)	+28.0%	0.64	0.0898	0.1098
Calf muscle AS score	2 (2; 3)	2 (1; 2)	−0.89 (−1.00 to −0.67)	−36.4%	−1.00	0.0078	0.0172
Walking speed (m/s)	0.79 (0.26)	0.90 (0.25)	0.11 (0.07 to 0.15)	+14.0%	1.00	0.0039 *	0.0172
Cadence (stride/min)	53.3 (9.5)	53.3 (8.9)	0.0 (−3.9 to 2.6)	0.0%	0.16	0.7109	0.7820
Affected-limb single-support (% gc)	31.5 (2.7)	33.8 (2.8)	2.3 (1.2 to 3.3)	+7.3%	0.96	0.0078	0.0172
Affected-limb single-support (s)	0.36 (0.03)	0.38 (0.04)	0.027 (0.011 to 0.054)	+7.5%	1.00	0.0313	0.0430
Affected-limb stride length (m)	0.90 (0.22)	0.96 (0.21)	0.066 (0.049 to 0.089)	+7.3%	1.00	0.0039 *	0.0172
Double-support time (% gc)	31.5 (9.5)	28.6 (7.8)	−2.86 (−5.83 to −1.24)	−9.1%	−0.96	0.0078	0.0172
Double-support time (s)	0.41 (0.23)	0.31 (0.11)	−0.101 (−0.221 to −0.040)	−24.6%	−0.87	0.0195	0.0307
Affected-limb stance phase (% gc)	63.0 (7.0)	62.4 (5.5)	−0.53 (−2.71 to 0.49)	−0.8%	−0.07	0.9102	0.9102

Values are presented as mean (SD), except for the AS score, which is presented as median (IQR). Changes are mean paired differences with bias-corrected and accelerated bootstrap 95% confidence intervals (CI). *r*rb, matched-pairs rank-biserial correlation. Positive *r*rb values indicate an increase from T0 to T1, whereas negative values indicate a decrease. FDR *q*-values were calculated using the Benjamini–Hochberg procedure across 11 outcomes. * Statistically significant after Bonferroni correction, with α = 0.05/11 = 0.0045

## Data Availability

The raw data supporting the conclusions of this article will be made available by the authors on request.

## Abbreviations

The following abbreviations are used in this manuscript:

CP	Cerebral Palsy
BoNT/A	Botulinum Toxin Type A
PROM	Passive Range of Motion
AROM	Active Range of Motion
AS	Ashworth Scale
